# Hydrogel‐Reactive‐Microenvironment Powering Reconfiguration of Polymer Architectures

**DOI:** 10.1002/advs.202307830

**Published:** 2024-04-08

**Authors:** Pengchao Liu, Zhengyi Mao, Yan Zhao, Jian'an Yin, Chengshengze Chu, Xuliang Chen, Jian Lu

**Affiliations:** ^1^ Department of Mechanical Engineering City University of Hong Kong Hong Kong China; ^2^ CityU‐Shenzhen Futian Research Institute Shenzhen China; ^3^ Centre for Advanced Structural Materials City University of Hong Kong Shenzhen Research Institute Greater Bay Joint Division Shenyang National Laboratory for Materials Science Shenzhen China; ^4^ State Key Laboratory of Advanced Design and Manufacturing for Vehicle Body College of Mechanical and Vehicle Engineering Hunan University Changsha 410082 China; ^5^ Laboratory of Nanomaterials & Nanomechanics City University of Hong Kong Hong Kong China

**Keywords:** 3D printing, cellular architecture, chemical reaction, polymer lattice, reconfiguration

## Abstract

Reconfiguration of architected structures has great significance for achieving new topologies and functions of engineering materials. Existing reconfigurable strategies have been reported, including approaches based on heat, mechanical instability, swelling, origami/kirigami designs, and electromagnetic actuation. However, these approaches mainly involve physical interactions between the host materials and the relevant stimuli. Herein, a novel, easy‐manipulated, and controllable reconfiguration strategy for polymer architectures is proposed by using a chemical reaction of host material within a hydrogel reactive microenvironment. 3D printed polycaprolactone (PCL) lattices transformed in an aqueous polyacrylamide (PAAm) hydrogel precursor solution, in which ultraviolet (UV) light triggered heterogeneous grafting polymerization between PCL and AAm. In situ microscopy shows that PCL beams go through volumetric expansion and cooperative buckling, resulting in transformation of PCL lattices into sinusoidal patterns. The transformation process can be tuned easily and patterned through the adjustment of the PCL beam diameter, unit cell width, and UV light on–off state. Controlling domain formation is achieved by using UV masks. This framework enables the design, fabrication, and programming of architected materials and inspires the development of novel 4D printing approaches.

## Graphical abstract

A novel hydrogel‐reactive‐microenvironment powered reconfiguration strategy is proposed for polymer architectures and chemical‐reaction‐assisted 4D printing. Melt electrowritten PCL architectures are sealed within an aqueous solution containing AAm as monomer. UV irradiation triggers a heterogeneous graft polymerization between PCL and AAm monomer. Consequently, PCL beams go through large volumetric expansion, causing the reconfiguration of PCL architectures.

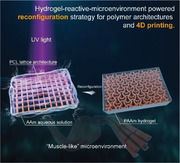

## 1 Introduction

Architected materials have attracted considerable attention owing to their excellent characteristics, including negative Poisson's ratio;^[^ ^1^, ^2^ ^]^ negative refractive index;^[^ ^3^, ^4^ ^]^ tunable thermal expansion;^[^ ^5^ ^]^ and coupled original conflicting material properties such as ultrahigh strength yet low weight,^[^ ^6^, ^7^, ^8^ ^]^ miniaturization yet high magnification,^[^ ^9^ ^]^ and ultralow thermal conductivity yet high stiffness.^[^ ^10^ ^]^ The reconfiguration of architected materials is vital for achieving new topologies and functions in the materials science and biological sectors. Interesting strategies that leverage swelling,^[^ ^11^, ^12^, ^13^, ^14^ ^]^ electromagnetic actuation,^[^ ^15^, ^16^, ^17^ ^]^ heat,^[^ ^18^ ^]^ liquid evaporation‐induced capillary force,^[^ ^19^ ^]^ origami/kirigami designs,^[^ ^20^, ^21^, ^22^ ^]^ and mechanical instabilities^[^ ^23^, ^24^, ^25^, ^26^ ^]^ have been reported to enable the shape‐morphing or deformation of architected materials. However, most of these reconfigurable systems feature complex material compositions, require complicated and expensive manufacturing procedures, or cannot be maintained without external stimulation. Moreover, the reported approaches mainly involve harnessing the physical interactions between the host materials and their relevant stimuli. Approaches based on the chemical reaction of host materials are rarely to be explored.

Hydrogel‐assisted additive manufacturing approaches, such as the freeform reversible embedding of suspended hydrogel method,^[^ ^27^, ^28^, ^29^ ^]^ the hydrogel infusion‐based approach for micro‐architected metals,^[^ ^30^ ^]^ and sacrificed hydrogel‐based 3D printing for channel structures,^[^ ^31^, ^32^ ^]^ have attracted increasing attention for engineering materials. However, these hydrogels have only been adopted as support materials or templates, without chemical reactive interactions with the host printing materials. The human body can be considered a complex hydrogel‐based matrix containing living cells and tissues.^[^ ^33^, ^34^ ^]^ Muscle fibers, which are key tissues in the human body, undergo reconfiguration by acquiring energy from the surrounding hydrogel matrix, and function normally for posture maintenance, locomotion, and control of various circulatory systems. The energy is derived from the chemical conversion of adenosine triphosphate to adenosine diphosphate.^[^ ^35^ ^]^

Inspired by the muscle fibers’ microenvironment (in the hydrogel matrix) and reconfiguration mechanism (contraction by acquiring chemical energy), we introduce a novel hydrogel‐reactive‐microenvironment powered reconfiguration mechanism to deform polymer architectures in their chemically reactive hydrogel precursor solution, a “muscle‐like” microenvironmental system. PCL and a PAAm hydrogel precursor solution (AAm as the monomer) were used as a prototype system. PCL is a linear aliphatic polyester with application in various biomedical tools, such as sutures,^[^ ^36^ ^]^ vascular grafts,^[^ ^37^ ^]^ and drug carriers.^[^ ^38^ ^]^ PCL can be initiated by radical polymerization and modified by crosslinking, and graft‐copolymerization under high‐energy irradiation, such as γ, electron beam, and X‐ray.^[^ ^39^ ^]^ These irradiation methods have possible destructive effects on polymers due to their high energy. PAAm hydrogel can be synthesized via the free radical polymerization of acrylamide (AAm) monomer under UV irradiation.^[^ ^40^ ^]^ UV light irradiation, with much lower energy, is chosen as the irradiation method to initiate the graft‐polymerization of PCL and AAm in the current study.^[^ ^41^ ^]^ Consequently, the originally hydrophobic PCL fibers swelled and expanded volumetrically, driving continuous, tunable, and irreversible structural reconfiguration of the polymer architectures. By using the proposed strategy, PCL lattices fabricated via melt electrowriting (MEW) exhibited topological reconfiguration and patterns. This study is the first to adopt a UV‐initiated grafting polymerization reaction as a design tool to achieve architectural reconfiguration. The approach is characterized by the simplicity of the material system, low‐cost manufacturing procedures, and noncontact operations; moreover, it can be extended to other reactive material systems and structures. In addition, our proposed approach inspires a novel 4D printing strategy assisted by chemical reactions.

## 2 Results and Discussion

### 2.1 Fabrication and Reconfiguration of PCL Architectures

**Figure 1:**
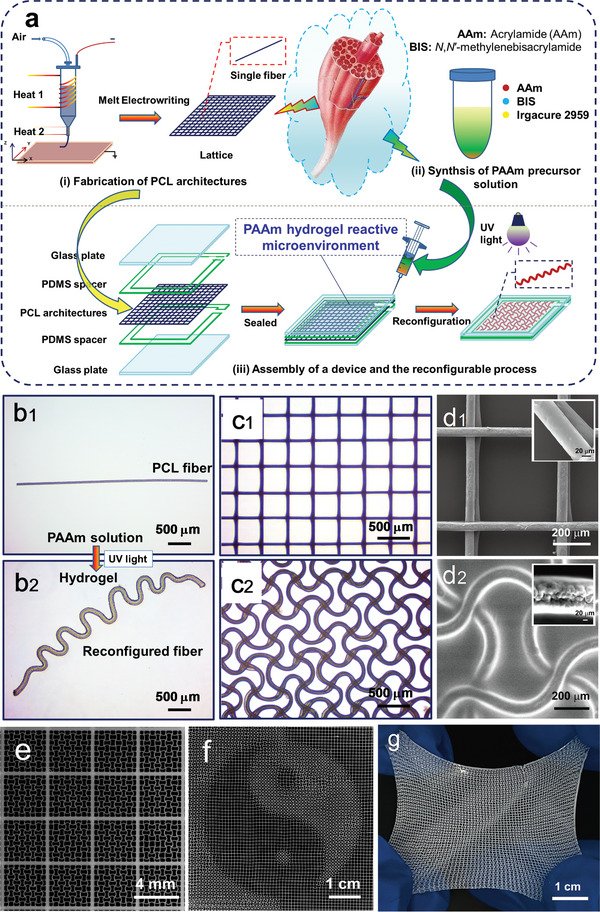
Fabrication, reconfiguration, and microscopy (optical and ESEM)‐based characterization of PCL architectures (fibers and lattices). a) Schematic illustration of fabrication and reconfigurable process of PCL architectures. Optical images of b_1_) a PCL fiber with a length of 2091.5 µm and b_2_) the corresponded PCL fiber after reconfiguration. Optical images of c_1_) PCL initial tetragonal lattices and c_2_) the corresponded PCL lattices after reconfiguration. ESEM images of a unit cell of d_1_) the initial tetragonal PCL lattice and d_2_) the reconfigured PCL lattice (insets: ESEM images of PCL beams before and after reconfiguration). b_1_,c_1_) PCL architectures embedded within the PAAm precursor solution; b_2_, c_2_, and d_2_) PCL architectures embedded within the PAAm hydrogel matrix). e) An optical image of PCL lattices with large area and low relative error after reconfiguration within the PAAm hydrogel matrix. f) An optical image of PCL lattices with a “Taiji” pattern after reconfiguration. g) An optical image of reconfigured PCL lattices within the transparent and elastic PAAm hydrogel matrix.

Our strategy is inspired by the locomotion behavior of muscle fibers by acquiring chemical energy from their surrounded microenvironment. PCL fibers and PCL lattices with tetragonal architectures were prepared via MEW,^[^ ^42^ ^]^ a special 3D printing approach based on melt electrospinning. A PAAm hydrogel precursor solution was prepared and used as the hydrogel reactive microenvironment to power the reconfiguration of the PCL architectures (i.e., fiber and lattices). PCL architectures were sealed within an self‐assembled device (**Figure** **1**a; Figure S1, Supporting Information) which was followed by the injection of PAAm hydrogel precursor solution. The reconfiguration process of PCL architectures was observed after UV exposure to the device. As a basic element of PCL lattice, PCL fiber's reconfiguration process was investigated first. A PCL fiber with a length of 2091.5 µm was prepared and placed in PAAm hydrogel precursor solution (Figure 1b_1_). No transformation of the PCL fiber was observed without UV exposure. After UV exposure, the fiber transformed from an initial linear state into a significant curved state by going through a large volumetric expansion: a radial growth of 100.5%, cross‐sectional area increases of 488%, and axial elongation of 143%. Simultaneously, PAAm precursor solution turned into PAAm hydrogel, where the reconfigured PCL fiber was embedded (Figure 1b_2_). A tetragonal PCL lattice contained eight layers of PCL beams with diameter of ≈48 µm and a unit cell width of 500 µm (Figures 1c_1_,d_1_). Similar treatment to PCL fiber, the PCL lattice went through a reconfiguration process from an initial tetragonal lattice into a sinusoidal pattern via cooperative beam curving, causing pairwise opposite concavity (Figures 1c_2_,d_2_). The PCL beams were bent to induce torque, and each pair of neighboring nodes rotated in opposite directions. Four PCL beams, which converged at a specific node, buckled in the coupled directions owing to the node rotation, consequently forming ordered sinusoidal patterns (Figure 1c_2_). Movie S1 (Supporting Information) showed the dynamic deformation process of a tetragonal PCL lattice. PCL beams presented volumetric expansion and cracks on the surface after reconfiguration (inset, Figure 1d_2_). The deformed PCL lattice was also embedded within the PAAm hydrogel matrix. Previous studies designed and fabricated polymer lattices at a scale of several tens of micrometers using photolithography.^[^ ^15^ ^]^ We are able to fabricate reconfigured lattices with a scale of tens of centimeters (Figure 1e). The reconfiguration domains were controlled in specific zones and form patterns by using UV masks. The “Taiji” pattern was formed by selectively covering areas on the PCL lattices (Figure 1f), as discussed in the following text. Figure 1g presented a reconfigured PCL lattice within the matrix of transparent and highly elastic PAAm hydrogel.

### 2.2 The Hydrogel‐Reactive‐Microenvironment Powered Strategy and Its Reconfiguration Mechanism

Previous studies found that PCL could be modified under high‐energy radiation (i.e., γ, electron beam, and X‐ray).^[^ ^39^ ^]^ However, few studies showed that PCL could be modified under UV light‐a moderate‐energy radiation. Original PCL cannot be swollen in an aqueous solution owing to its hydrophobicity.^[^ ^43^ ^]^ To explain the mechanism responsible for the reconfiguration of the PCL lattices, PCL beams with initial lengths of 348.7, 704.0, and 2091.5 µm were prepared. As the basic element of the PCL lattice, the chosen PCL beams were subjected to the same treatment (immersed in PAAm precursor solution + UV light exposure) as in the PCL lattice reconfiguration process.

**Figure 2:**
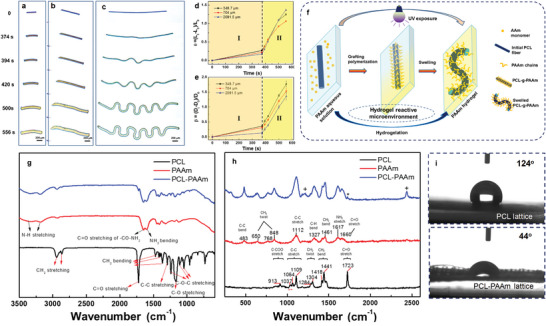
A proposed mechanism for the reconfiguration mechanism of the hydrogel‐reactive‐microenvironment powered strategy. a, b, and c) Deformation process versus UV light exposure time of PCL beams with lengths of 348.7, 704.0, and 2191.5 µm, respectively. d) The axial length elongation ratio and e) radial expansion ratio of PCL beams versus UV light exposure time (*θ* denotes the axial length elongation ratio; *L*_0_ and *L_t_* denote the PCL beam lengths at UV exposure times of 0 and *t* s, respectively; *β* denotes the radial expansion ratio; and *D*_0_ and *D_t_* denote the PCL beam diameters at UV exposure times of 0 and *t* s, respectively.) f) Schematic of the proposed mechanism of the hydrogel‐reactive‐microenvironment powered strategy. Stage I: UV light irradiation of the PCL beam surface in the PAAm hydrogel precursor solution; the volumetric expansion of the PCL beams is negligible. Stage II: The volumetric expansion of the PCL beams increased rapidly with irradiation time in both the axial and radial directions owing to the graft polymerization between PCL and the AAm monomer and further swelling by AAm and water infiltration into the matrix of the PCL beams. g) FTIR and h) Raman spectra of the PCL lattice, the PAAm hydrogel matrix, and the reconfigured PCL–PAAm lattice. i) Images of static water contact angles on the PCL and PCL–PAAm lattices.

We constructed an in situ optical setup (Figure S1, Supporting Information) to capture real‐time snapshots of the reconfiguration process of the PCL beams and lattices. All PCL beams exhibited obvious volumetric expansion in radial and axial directions (**Figures** 2a–c). The final status of the PCL beam with a length of 348.7 µm exhibited a curved “banana shape” (Figure **2**a), similar to the findings of a previous study in which SU‐8 banana‐shaped particles were synthesized.^[^ ^18^ ^]^ The PCL beam with a length of ≈704.0 µm exhibited a bidirectional curvature (Figure 2b). The PCL beam with a length of 2091.5 µm underwent volumetric expansion to transform from the initial linear state into a more significant curved state (Figure 2c). Movies S2 and S3 (Supporting Information) present the volumetric expansion and curvature process of the PCL beams with lengths of 348.7, 704.0, and 2091.5 µm. The significant volumetric expansion of the PCL beam was responsible for the lattice reconfiguration. Interestingly, volumetric expansion of a silicon electrode material after lithiation enabled the reconfiguration of silicon–lithium architectures.^[^ ^15^ ^]^ However, their electrochemical reconfiguration strategy was by exploiting alloying/dealloying reactions and only suitable for alloy system.

The length and diameter of the PCL beams exposed to UV light were statistically analyzed. The measured length and diameter of the PCL beams, calculation formulas, and methods for calculating the expansion ratio of the PCL lattice are shown in Figure S2 (Supporting Information). The deformation process of PCL beams can be divided into two stages (Figures 2d,e): In Stage I (UV exposure time: 0–396 s), the expansion of PCL beams was almost negligible in both the axial and radial directions; in Stage II (UV exposure time: > 396 s), the PCL beams expanded rapidly in both the axial and radial directions. These two distinct stages during the reconfiguration process are also depicted in Movies S2 and S3 (Supporting Information).

Heterogeneous graft polymerization of polymers occurs in their solid state by contacting an aqueous monomer solution.^[^ ^39^, ^44^ ^]^ This chemical strategy is a versatile surface modification approach for hydrophilization of polymers. UV irradiation is commonly used for the introduction of free radicals of polymer, initiating the polymerization of the monomers attached to the polymer's surface to form grafted chains.^[^ ^41^, ^45^, ^46^ ^]^ The PCL beam, the PAAm hydrogel matrix, and the reconfigured PCL–PAAm beam were characterized via Fourier‐transform infrared (FTIR) spectroscopy. The FTIR spectra exhibited characteristic absorption bands of PCL and PAAm (Figure 2g), consistent with the findings in the literature.^[^ ^40^, ^47^, ^48^, ^49^ ^]^ The spectrum of the PCL–PAAm sample exhibited peaks at ≈1163 cm^−1^ (C─O stretching), 1721 cm^−1^ (C═O stretching), 2943 cm^−1^, and 2862 cm^−1^ (─CH_2_ stretching), which correspond to PCL, while the peaks at ≈1653 cm^−1^ (C═O stretching of ─CO─NH_2_) and 1595 cm^−1^ corresponded to PAAm. These results prove the occurrence of a heterogeneous grafting polymerization reaction between solid PCL and the AAm monomer in an aqueous solution under UV light. Raman spectroscopy, whose spectra are not affected by water, is suitable for characterizing PCL in the wet PAAm hydrogel matrix. The peaks of pure PCL and PAAm hydrogel peaks (Figure 2h) were consistent with the findings in the literature.^[^ ^43^, ^50^ ^]^ The Raman spectrum of PCL‐PAAm exhibited new peaks at 1213 and 2436 cm^−1^, and the characteristic peak at 1723 cm^−1^, corresponding to PCL, was significantly weakened compared with that of the PCL spectrum. These results also substantiate the occurrence of heterogeneous grafting polymerization between PCL and AAm.

The grafting polymerization reaction between PCL and AAm can be achieved by simultaneous grafting with polymerization or by using a preirradiation approach.^[^ ^39^ ^]^ Our current study adopted the former method. The mechanism and process can be described like this: UV light irradiated the surface of PCL fibers/lattices. Irradiation initiated the formation of secondary alkylether or peroxy radicals.^[^ ^51^ ^]^ AAm monomer is not only hydrophilic but also highly reactive. AAm monomer reacted with radicals on the surface of PCL substrates, accompanying the synthesis of PAAm chain due to the homopolymerization of AAm. As a result, the graft polymerization of PCL‐g‐PAAm occurred, achieving grafting PAAm chain onto the PCL substrate from surface to bulk.^[^ ^43^ ^]^

Our experimental results agree with those of Albertsson's group, who found that a PCL film preirradiated by electron beams could be graft‐polymerized with AAm as a monomer in an aqueous solution, resulting in volumetric expansion of PCL film.^[^ ^51^, ^52^ ^]^ The researchers further built a multi‐layer model of water diffusion in AAm‐grafted aliphatic polyesters, including PCL.^[^ ^53^ ^]^

The static water contact angles of the pure PCL lattice and the PCL–PAAm lattice were 124° and 44°, respectively (Figure 2i). This proves that the hydrophobic PCL surface became hydrophilic, which proved further the occurrence of graft‐polymerization of PCL and AAm.

According to the above discussion, the hydrogel‐reactive‐microenvironment powered reconfiguration mechanism of the PCL lattice is described as follows (Figure 2f): UV light irradiated the surfaces of the PCL beams (Stage I) and further resulted in graft polymerization^[^ ^48^, ^54^ ^]^ between PCL and the AAm monomer in the PAAm precursor solution (Stage II). UV‐initiated graft‐polymerization led to a change of PCL in surface property from hydrophobic to hydrophilic, accompanying the hydrophilic AAm monomers’ penetration into the grafting front within the PCL matrix and swollen the PCL. Eventually, these elements caused the volumetric expansion of PCL. The volumetric expansion was restrained by the structure of the nodes, the fixed edges of the PCL lattice, and the hydrogelation‐induced increasing viscosity of the PAAm precursor solution. PCL beams expand greatly and buckle to reduce the generated stress under the constraints. To minimize the generated stress, PCL beams tend to bend bidirectional, causing cooperative buckling of PCL beams and reconfiguration of PCL lattice.

To test our strategy, a triangular PCL lattice was fabricated via MEW and then placed in a PAAm precursor solution. The lattice was exposed to UV light, and the deformation process was observed (Figure S3 and Movie S4, Supporting Information). Notably, such reconfigurable process is not specific to the PCL–PAAm chemistry. A tetragonal polylactic acid (PLA) lattice fabricated via MEW exhibited similar beams’ expansion and cooperative buckling in the PAAm precursor solution under UV light (Figure S4 and Movie S5, Supporting Information). Rahman et al. highlighted that UV irradiation‐induced a surface grafting reaction between PLA films and the AAm monomer.^[^ ^55^, ^56^ ^]^

### 2.3 Effect of Lattice Parameters on Reconfiguration

**Figure 3:**
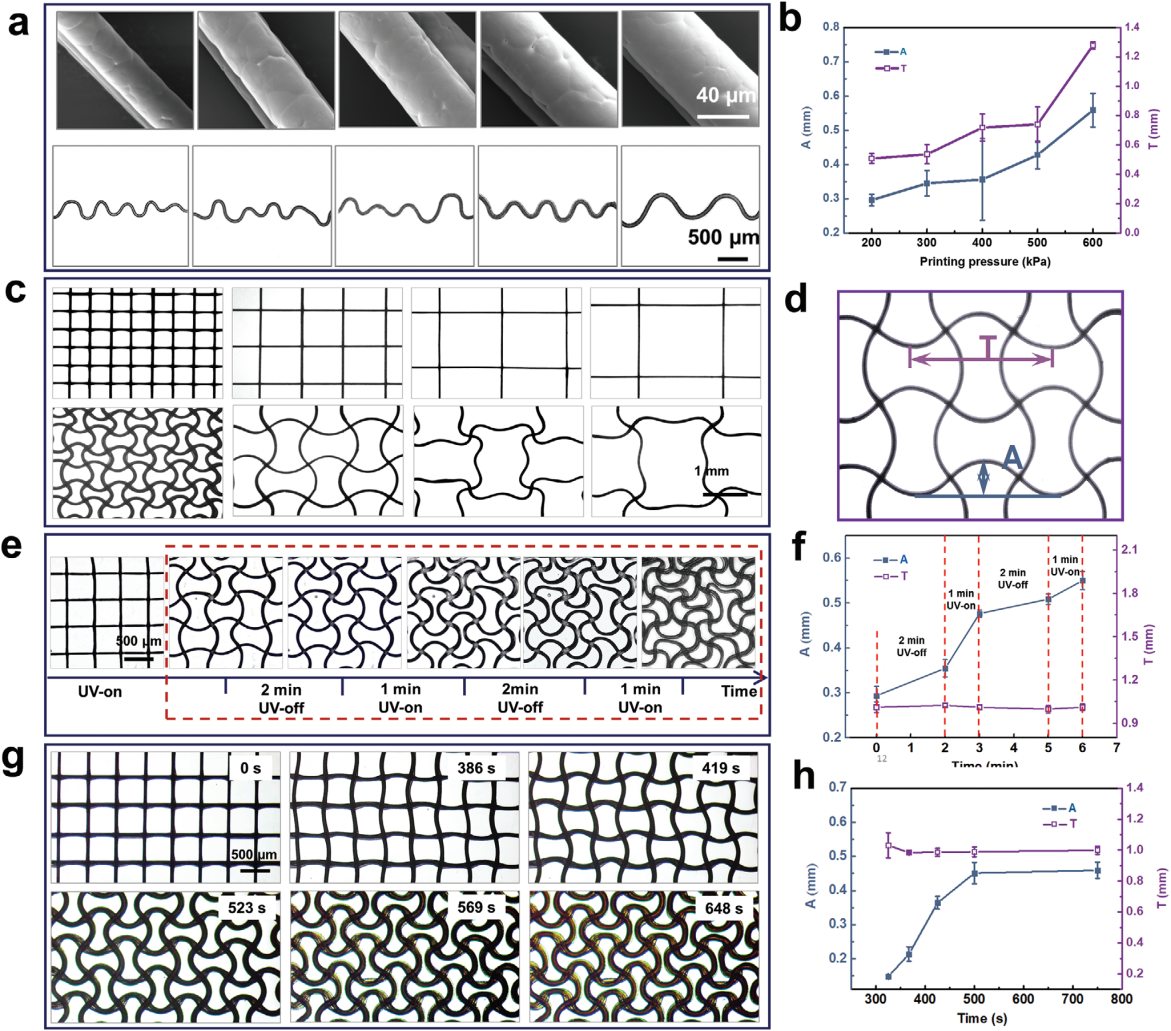
Tuning and controlling the deformation of PCL lattice architecture. a) Images of PCL beams with diameters of ≈35 ± 4.3 µm (200 kPa), 40 ± 5.6 µm (300 kPa), 48 ± 3.0 µm (400 kPa), 51 ± 4.2 µm (500 kPa), and 56 ± 4.0 µm (600 kPa) and the corresponding ones after reconfiguration. c) Tetragonal PCL lattices with unit cell widths of 0.50, 1.00, 1.50, and 2.00 mm and the corresponding ones after reconfiguration. e) Effect of the UV on‐off state on the deformation processes of tetragonal PCL lattices. The architectures rapidly deformed under UV exposure, and the deformation was negligible in the UV‐off state. g) Effects of UV exposure time on the deformation of tetragonal PCL lattices. d) Amplitude (*A*) and wavelength (*T*) measurements of the sinusoidal patterns. b, f,h) are plots of *A* and *T* versus deformation time, corresponding to (a), (e), and (g), respectively.

The volumetric expansion of PCL beams is the driving force for the lattice reconfiguration. The structural parameters of PCL architectures can influence the reconfiguration process. The effects of the PCL beam diameter and the unit cell width of the PCL lattice on the wavelength (*T*) and amplitude (*A*) (Figure 3d) were investigated to quantitatively characterize the topological reconfiguration process.

PCL beams are the basic element of the PCL lattice. Their diameter determines the swollen extent of the beams and further whether the volumetric expansion is powerful enough to drive the reconfiguration of PCL lattice. We fabricated PCL beams (**Figure** 3a) and lattices (Figure S5, Supporting Information) under extrusion pressures of 200–600 kPa. The corresponding diameters of the PCL beams were 35 ± 4.3 µm (200 kPa), 40 ± 5.6 µm (300 kPa), 48 ± 3.0 µm (400 kPa), 51 ± 4.2 µm (500 kPa), and 56 ± 4.0 µm (600 kPa) (Figure S6, Supporting Information). In Figure 3a and Figure S7 (Supporting Information), the number of curves decreases with an increasing *A* as the beam diameter increases. This indicates that the driving force of volumetric expansion was weaker with increasing diameter. *T* and *A* increased with increasing diameter (Figure 3b), indicating the possibility of tuning the reconfigured architecture through extrusion pressure adjustment during the MEW process.

Unless otherwise specified, the following text refers to the experiment on the tetragonal PCL lattice fabricated at 400 kPa. The unit cell width determined the node density in the lattice, which was another key factor influencing the resistance to volumetric expansion. A smaller unit cell width (a larger number of nodes) corresponds to a higher resistance. Ordered sinusoidal patterns were generated at a unit cell width of < 1.50 mm (Figure 3c). In contrast, architectures with unit cell widths of 1.50 and 2.00 mm exhibited more than one wave in each lattice unit. Thus, PCL lattices with tailored patterns can be fabricated via appropriate unit cell width adjustment.

The UV light on–off state and exposure time are critical factors influencing the deformation process of the PCL lattice. The tetragonal PCL lattice with a unit cell width of 0.50 mm was used in the corresponding experiment. The UV light was switched on or off to observe the deformation process. The control program was set as follows: 2 min (off)–1 min (on)–2 min (off)–1 min (on) in Stage II of the deformation process (Figure 3e). Movie S6 (Supporting Information) clearly showed that nearly no deformation was observed under the UV‐off status, whereas deformation rapidly continued under the UV‐on status. As can be seen in Figure 3f, *A* increased slightly over time under the UV‐off status, whereas increased rapidly under the UV‐on status. *T* was stable throughout the process owing to the constraint. The deformation phenomenon could be interrupted and resumed, indicating that the geometries during the deformation could be captured via radiation mode control. By maintaining the UV‐on status, we found that the PCL lattice deformation was time‐dependent (Figure 3g; Movie S1, Supporting Information). During the deformation process, *T* was stable owing to the constraint, and *A* increased rapidly with the exposure time (Figure 3h).

### 2.4 Finite‐Element Analysis of the Reconfiguration Process in Stage II

**Figure 4:**
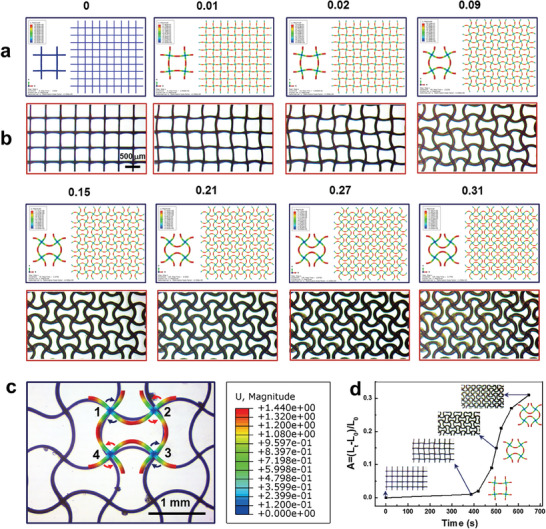
Chemo‐mechanical FEA modeling of tetragonal PCL lattices. a) Simulated and b) experimental PCL lattice geometries with different axial elongation ratios over the deformation period. c) FEA simulation results of a unit cell, overlaid onto optical images. d) Experimental axial elongation ratio versus time over the deformation period.

A nonlinear finite‐element analysis (FEA) model^[^ ^57^ ^]^ was used to quantitatively investigate the topological reconfiguration process and elucidate the mechanical behavior of the PCL lattice in Stage II. The lattices were considered to be composed of a neo‐Hookean hyperelastic material, which could account for large elastic deformations. **Figure** **4**a shows snapshots of the simulated PCL lattice geometries with different axial length elongation ratios, captured from the deformation process recording in Movie S1 (Supporting Information). Movie S7 (Supporting Information) shows the geometry evolution of tetragonal PCL lattices, summarizing the simulation results. When the expansion strain exceeded a critical value, the lattices exhibited Euler buckling. *A* increased with the increasing expansion strain. The results shown in Figures 4a,b demonstrate the ability of the FEA model to quantitatively predict the mechanical behavior of the PCL lattice.

The finite‐element simulation results for a deformed unit cell were compared with an experimental optical image showing the typical ordered sinusoidal patterns of the deformed tetragonal PCL lattice (Figure 4c). In a typical unit cell, nodes 1 and 3 rotated clockwise, whereas nodes 2 and 4 rotated anticlockwise. The simulation results were consistent with the experimental data and reflected the mechanical behaviors in terms of the rotation directions of the nodes, beam curving directions, and coupled buckling propagation characteristics. All adjacent nodes on the same horizontal plane rotated in opposite directions, and all beams formed coherent sinusoidal lines with pairwise opposite curved surfaces. The simulated images (Figure 4a) and deformation process video (Movie S7, Supporting Information) were consistent with the experimental entities. This demonstrates that the FEA model can be applied to predict mechanical behaviors. The PCL lattice transformation process featured two distinct stages (Figure 4d). The deformation process of the triangular PCL lattice structure was also simulated via FEA (Figure S8 and Movie S8, Supporting Information). The results demonstrate the quantitative prediction ability of the proposed FEA model.

### 2.5 Control of Domain Formation Using UV Masks

The experimental results indicate that volumetric expansion led to the cooperative buckling of PCL beams in the lattice, and that the beams formed identical sinusoidal patterns during PAAm hydrogel gelation under UV light. As earlier demonstrated, UV light plays a vital role in controlling the volumetric expansion and buckling of the PCL beam. For example, in Stage II, PCL beams buckled under UV light. Thus, specific buckling configurations can be designed and fabricated through selective shielding of the PCL lattice zones under UV light.

**Figure 5:**
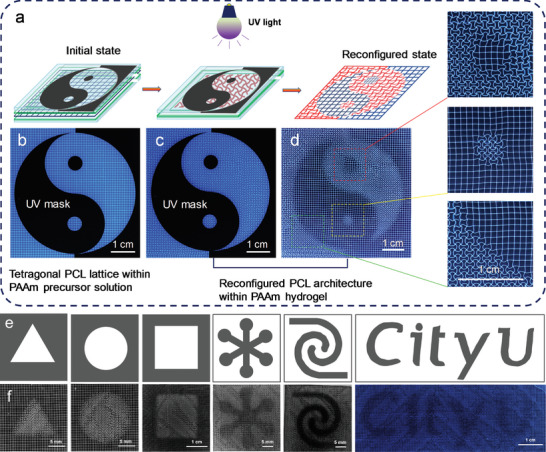
Controlling domain reconfiguration through UV masks. a) Schematic illustration of the controlling domain reconfiguration with a “Taiji”‐shaped UV mask as a model. Images of b) a tetragonal PCL lattice within PAAm precursor solution and c) the reconfigured PCL lattice within PAAm hydrogel, covered by a UV mask with a “Taiji” pattern. d) An image of “Taiji” pattern after removing the UV mask. e) Schematic diagrams of UV masks: White zones represent transparent polyethylene terephthalate, which is penetrable by UV light. Grey zones represent black polyethylene terephthalate, which is impenetrable by UV light. f) Optical images of experimental architectures after programmed deformation. (The CityU icon is used with permission.).

To control the PCL beam transformation in specific zones and form patterns, we designed and prepared UV masks (Figure S9, Supporting Information). The masks were placed on the sealed device during the experiment, and the sample was irradiated by overhead UV light. A “Taiji”‐shaped UV mask was chosen as a model to illustrate the formation process of pattern by controlling domain reconfiguration (**Figure** 5a). The UV light penetrated the transparent zone of the mask (Figure 5b), triggering the volumetric expansion and buckling of the PCL beams (Figure 5c). However, the polymer architectures under the mask shade retained their initial state, as the mask blocked UV light. Finally, a “Taiji” pattern was obtained after removing the UV mask (Figure 5d). By designing various UV masks as shown in Figure 5e, triangular, round, square, snowflake‐shaped, hyperbolic, and “City U” patterns were fabricated (Figure 5f). In addition, complementary patterns can be also obtained by designing complementary UV masks (Figure S10, Supporting Information). Diverse patterns can be formed by controlling domain reconfiguration of PCL architectures. This finding endows a potential application in information steganography (Figure S11, Supporting Information).

**Figure 6:**
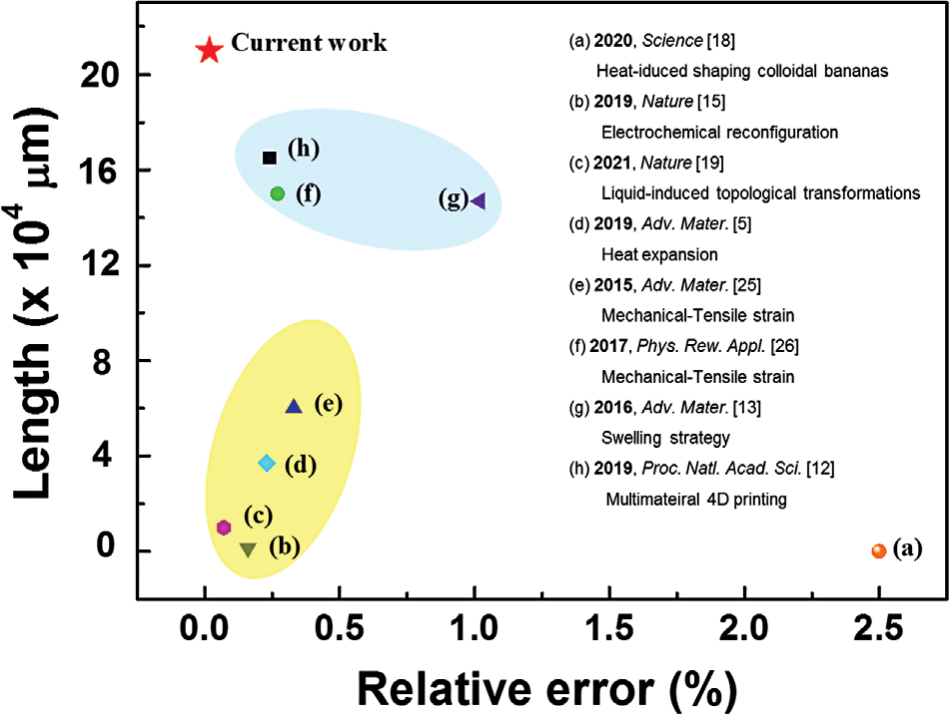
Comparison of the hydrogel‐reactive‐microenvironment reconfigured polymer architectures with large area and low relative error (red star) with other reconfiguration strategies. Length denotes the lattice length; relative error is the ratio of the beam diameter or plate thickness to the lattice length.

It is difficult to simultaneously achieve large area and high printing accuracy in 3D/4D‐printed architected materials. Undoubtedly, engineering materials will be applied more widely with these two advantages combined. “Low relative error” is a more meaningful phrase than “high printing accuracy”. Here, the relative error was defined as the ratio of the beam diameter to the lattice length. In our system, we obtained a PCL lattice with a relative error of 0.017% (PCL beam diameter: 35 µm [Figure S6, Supporting Information], PCL lattice length: 21 cm [Figure S14, Supporting Information]). It is worth noting that the relative error can be further decreased in our system by reducing the printing nozzle's diameter or the extrusion pressure. A comparison of our strategy with previously reported architecture reconfiguration approaches (**Figure** **6**; Table S1, Supporting Information) revealed that the hydrogel‐reactive microenvironment powered strategy can simultaneously achieve large area and low relative error.

## 3 Discussion

The novel hydrogel‐reactive microenvironment powered strategy involves the UV light‐initiated graft polymerization of a polymer with the monomer of a hydrogel. Its mechanism is different from those of the existing reconfiguration approaches^[^ ^11^, ^12^, ^13^, ^14^, ^15^, ^16^, ^17^, ^18^, ^19^, ^20^, ^21^, ^22^, ^23^, ^24^, ^25^, ^26^ ^]^ based on physical interactions between the host materials and the stimuli. In this study, we built a chemical reactive field‐PAAm hydrogel precursor microenvironment in which 3D‐printed PCL fibers and lattices were placed. UV light triggered graft polymerization between PCL and AAm, endowing the originally hydrophobic PCL surface with hydrophilicity. Consequently, AAm monomer and water diffuse to the grafting front within the PCL matrix and cause it to swell, resulting in the volumetric expansion of PCL beams. The PCL beams expanded greatly and underwent buckling to reduce the generated stress under the constraints of the node structure and fixed PCL lattice edges. The cooperative buckling of PCL beams in the 3D‐printed lattice enabled reconfiguration from initial tetragonal or triangular geometries. The buckling degree could be continuously modulated through the adjustment of the PCL beam diameter, unit cell width, UV on–off state, and UV exposure time. Different patterns were fabricated through the selective shielding of UV light‐exposed zones with UV masks. The current strategy provides a novel chemical reaction‐based platform for establishing macroscale reconfigurable architected materials with a low relative error.

The proposed strategy is also a potential 4D printing method. Light plays a key role in 3D/4D printing processes. Light‐assisted 3D printing, in the form of digital light processing and stereolithography, is based on monomer polymerization under UV light or a laser beam.^[^ ^58^ ^]^ In these 3D printing process, light usually does not trigger structural transformation but only provides the energy to initiate radical polymerization. In light‐responsive 4D printing, heat from the light source leads to a change in the geometry of shape‐memory materials.^[^ ^59^ ^]^ The mechanical transformation is not a direct result of the light energy but is triggered by the light‐induced heat. The heat can cause the temperature of the materials to exceed the glass‐transition temperature of shape‐memory polymers or the phase transition temperature of shape‐memory alloys. The proposed 4D printing strategy in our current study is different from both light‐assisted 3D printing and light‐responsive 4D printing.

Overall, we developed a novel and simple strategy to design and fabricate reconfigurable architectures via graft‐polymerization‐induced volumetric expansion. The hydrogel‐reactive microenvironment powered strategy can be extended to other similarly reactive material systems.^[^ ^45^, ^60^ ^]^ In addition, this framework can promote the design, manufacturing, prediction, and programming of reconfigurable architected materials, which can be potentially used in information steganography (Figure S11, Supporting Information), UV‐responsive smart glass (Figure S12, Supporting Information), and biomedical devices (e.g., systems for the interferometric profiling of extracellular vesicles,^[^ ^61^ ^]^ controlled drug‐release systems [Figure S13, Supporting Information]).

## 4 Experimental Section

### Fabrication of PCL fibers and Lattices

PCL fibers and lattices with tetragonal and triangular architectures were designed and fabricated using a MEW unit installed on the bioprinting platform (GeSiM Bioscaffolder 5.1, Grosserkmannsdorf, Germany). PCL pellets (Sigma–Aldrich, Mw = 45 000) were placed in stainless cartridges (125 °C) with a stainless nozzle (an inner diameter of 250 µm, 70 °C) for 1 h to form a homogeneous polymer melt. The distance between the nozzle and the collector, the layer height between two adjacent layers, and the voltage were set as 5 mm, 0.01 mm, and 10 kV, respectively. PCL beams were extruded through the nozzle at compressed air pressures of 200–600 kPa and deposited onto a plastic collector at a writing speed of 150 mm^ ^s^−1^. For each PCL lattice sample, eight layers of PCL beams were fabricated. The distance between adjacent beams was 500 µm. The obtained PCL lattices were removed from the collector for subsequent use.

### Preparation of the Aqueous PAAm Precursor Solution‐the Hydrogel Reactive Microenvironment

AAm, *N*,*N*′‐methylenebisacrylamide (BIS), and 2‐hydroxy‐4′‐(2‐hydroxyethoxy)−2‐methylpropiophenone (Irgacure 2959) were purchased from Sigma–Aldrich and used without any purification. The PAAm precursor solution, as the chemical reactive field for the PCL architectures reconfiguration, was a mixture of 100 µL (0.1 mol L^−1^) BIS aqueous solution, 50 µL (0.1 mol L^−1^ in ethanol) Irgacure 2959 solution, and 10 mL (30 wt%) AAm aqueous solution. After homogeneous mixing, the PAAm hydrogel precursor was stored away from light until further use.

### Curvature Generation and Volumetric Expansion of PCL fibers in PAAm Precursor Solution

PCL fibers were prepared via MEW and cut into sections of different lengths and placed on the surface of a 50 mm × 50 mm × 0.5 mm glass plate with a 200‐µm‐thick polydimethylsiloxane (PDMS) spacer. Another PDMS spacer and an upper glass plate with the same thickness were introduced successively onto the former spacer, resulting in a 400‐µm‐thick space for the experiment. Four edges of glass plates were clamped to prevent leakage of the PAAm precursor solution, which was injected into the space between two glass plates through a reserved gap of PDMS spacers. Finally, the assembled device was sealed with the PCL fibers and the PAAm precursor solution was exposed to UV light (365 nm, 10%, XM230, Shanghai Aventk Co. Ltd.). To observe the curvature generation and volumetric expansion process, three PCL fibers with lengths of 348.7, 704, and 2091.5 µm were selected.

### Reconfiguration of PCL Lattices in PAAm Precursor Solution

The reconfiguration process of the PCL lattices was similar to that of the PCL fibers. Specifically, the square‐shaped PCL lattices were placed in the sealed space between two glass plates. The four edges of the PCL lattice were fixed by the clamped glass plates, and then a PAAm precursor solution was injected into the sealed space. The PCL lattices were immersed in the PAAm precursor solution. Finally, the UV light source was placed directly above the sealed glass device and switched on to trigger the lattice reconfiguration.

### FEA Simulation

To investigate the mechanical behavior during chemical reaction‐induced buckling, FEA simulations were performed using the commercial software ABAQUS (2016). In the FEA model, the lattice dimensions were the same as those used in the experiments. Beam elements were used in the FEA model because the printing beam exhibited a large slenderness ratio. The lattice was considered to be a neo‐Hookean hyperelastic material during analysis, which could account for large elastic deformations. The shear modulus of the PCL was set as 22 MPa. Simply supported boundary conditions were imposed on the lattice structure borders. A pseudo‐dynamics algorithm was used to accurately reflect the buckling behavior of the structures.

### Characterization

Snapshots of the deformation process of the PCL beams and lattices were acquired using an optical microscope (Nikon, Eclipse TS100) The morphology of the PCL lattice after deformation in the PAAm hydrogel matrix was observed via environmental scanning electron microscopy (SEM, FEI Quanta 250). The diameters of the PCL beams were measured using the image analysis software ImageJ 1.40G (http://rsb.info.nih.gov/ij/download.html). The SEM images of at least 20 randomly selected samples were analyzed to obtain diameter distribution histograms. The FTIR spectra of the samples were recorded using the NEXUS‐8700 V infrared spectrophotometer (Thermo Electron Company, USA) at the wavelength range of 400–4000 cm^−1^. The Raman scattering spectra of the samples were obtained using a Raman system (WITec RAMAN alpha300R) with an argon ion laser (532 nm) as the excitation source. All spectra were recorded without specific polarization configuration, and the resolution of the spectrometer was 0.5 cm^−1^.

## Conflict of Interest

The authors declare no conflict of interest.

## Author Contributions

P.L. and Z.M. contributed equally to this work. J.L. and P.L. conceived and designed this work. P.L. and Z.M. conducted the fabrication, measurements, and analysis. Y.Z. contributed to the FEA simulation. J.Y. helped with the characterization of Raman spectroscopy. P.L. drafted the manuscript with the help of Z.M. and X.C. J.L. revised the manuscript and supervised the project. All authors contributed to the experimentation and discussion.

## Supporting information

Supporting Information

Supplemental Movie 1

Supplemental Movie 2

Supplemental Movie 3

Supplemental Movie 4

Supplemental Movie 5

Supplemental Movie 6

Supplemental Movie 7

Supplemental Movie 8

## Data Availability

The data that support the findings of this study are available in the supplementary material of this article.
