# Supplementary material for: Hydrogel‐Reactive‐Microenvironment Powering Reconfiguration of Polymer Architectures
Source: Adv Sci (Weinh). 2024 Apr 8;11(24):2307830. doi: 10.1002/advs.202307830 (PMC11199975; doi:10.1002/advs.202307830)
Supplement: Supplementary file 1 — Supporting Information [file ADVS-11-2307830-s007.pdf]

ADVANCED SCIENCE
Open Access

Supporting Information

for *Adv. Sci.*, DOI 10.1002/advs.202307830

Hydrogel-Reactive-Microenvironment Powering Reconfiguration of Polymer Architectures

*Pengchao Liu, Zhengyi Mao, Yan Zhao, Jian'an Yin, Chengshengze Chu, Xuliang Chen and Jian Lu**

Supporting Information

Hydrogel-Reactive-Microenvironment Powering Reconfiguration of Polymer Architectures

Pengchao Liu, Zhengyi Mao, Yan Zhao, Jian'an Yin, Chengshengze Chu, Xuliang Chen, Jian Lu*

This PDF file includes:

Figures S1 to S14
Table S1
Captions for Movies S1 to S8

Other Supplementary Materials for this manuscript include the following:

Movies S1 to S8

In situ optical microscopy setup

In situ optical microscopy setup included an optical microscope (Nikon, Eclipse TS100), an ultraviolet (UV) light resource (365 nm, 10%, XM230, Shanghai Aventk Co. Ltd.), and a tablet connected to the optical microscope. UV light was used to irradiate the sealed device embedded with PCL architectures and PAAm precursor solution and trigger the deformation of the polymeric lattice. The tablet was used to observe, take snapshots, and record videoes of the deformation process.

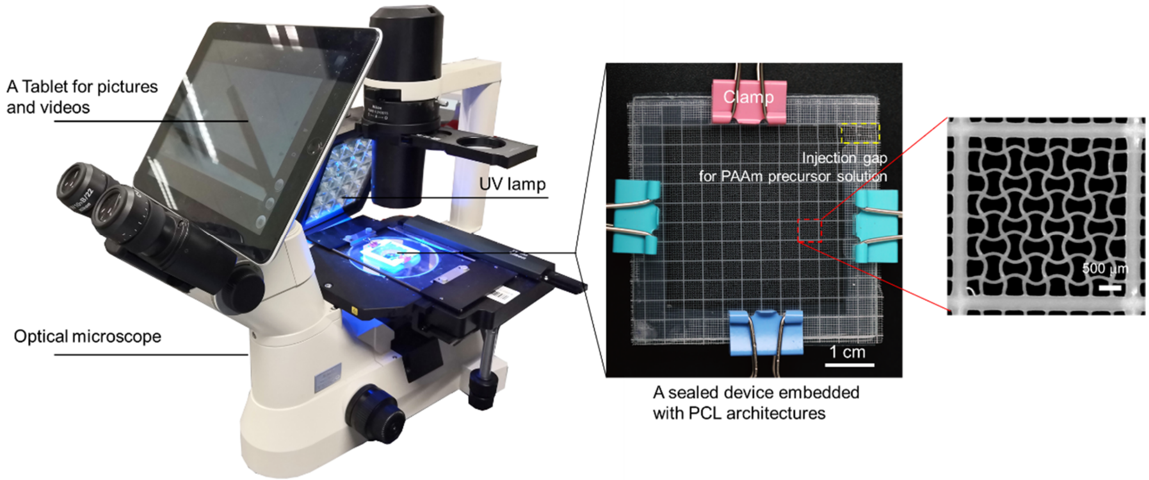

Figure S1. Images of the *in situ* optical microscopy setup and a sealed device embedded with a reconfigured PCL lattice (Figure 1e) and transparent PAAm hydrogel.

Statistical analysis of the expansion ratio of PCL beams and PCL lattices

The expansion ratio of the PCL beams, including the radial growth ratio and axial elongation ratio, was calculated. The relative lengths of the PCL beams exposed to UV light for different durations were measured using the image analysis software ImageJ 1.40G (http://rsb.info.nih.gov/ij/download.html). The axial length elongation ratio of the PCL beams was calculated using the following expression:

$$\boldsymbol{\theta = (L_t - L_0)/L_0} \qquad (1)$$

where θ represents the axial length elongation ratio, and L_0 and L_t represent the lengths of the PCL beams exposed to UV light for 0 and t s, respectively. The schematic of L_0 and L_t measurements is shown in Figure S2.

The radial expansion ratio of the PCL beams was calculated as

$$\boldsymbol{\beta = (D_t - D_0)/D_0} \qquad (2)$$

where $\boldsymbol{\beta}$ represents the radial expansion ratio, and D_0 and D_t represent the diameters of the PCL beams exposed to UV light for 0 and t s, respectively. The measurement procedures of D_0 and D_t were similar to those of L_0 and L_t.

The expansion ratio of the PCL lattice was calculated using Equation (1), in which L_0 and L_t represent the node X–Y lengths of the initial and deformed lattices, respectively. Equation (2) was not applied to calculate the expansion ratio of the PCL lattice because the lattice was composed of eight layers of the PCL beams, which may be misplaced after deformation, resulting in inaccurate diameter measurement.

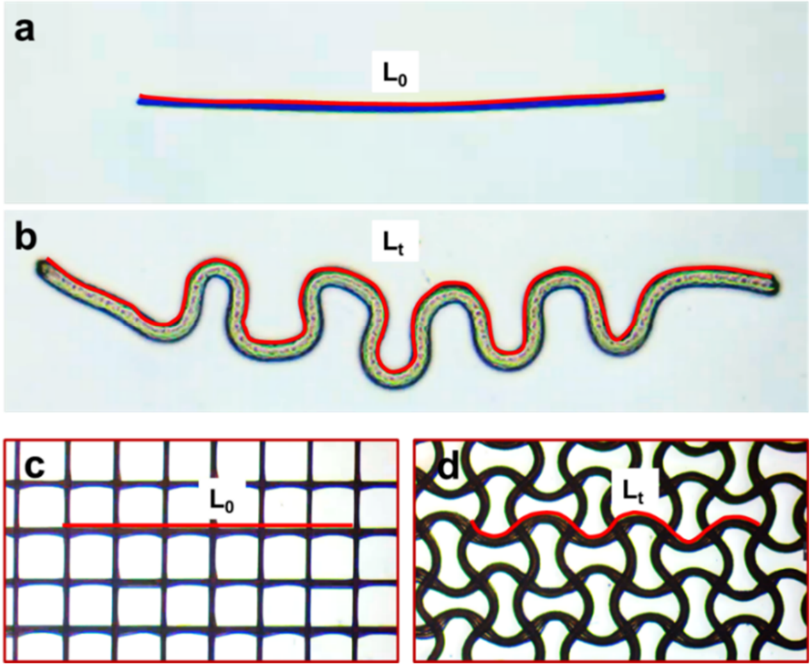

Figure S2. Schematics of length measurement for the initial and deformed (**a, b**) PCL beams and (**c, d**) PCL lattices for expansion ratio calculation. The red lines along the PCL beams were drawn and measured using the 'segmented line' tool in ImageJ 1.40G. For the tetragonal PCL lattice, the red lines were drawn along the outer edge of the PCL beams between two nodes. Formula (1) was used for calculation.

Fabrication of a triangular PCL lattice and its reconfigurability

A triangular PCL lattice was prepared via MEW. The fabrication process parameters were similar to those of the tetragonal PCL lattice. One triangular sample contained eight layers of PCL beams, and the distance between adjacent beams was 500 μm (Figure S3a). Under UV light, the original triangular lattice in the PAAm hydrogel precursor solution underwent deformation (Figure S3b).

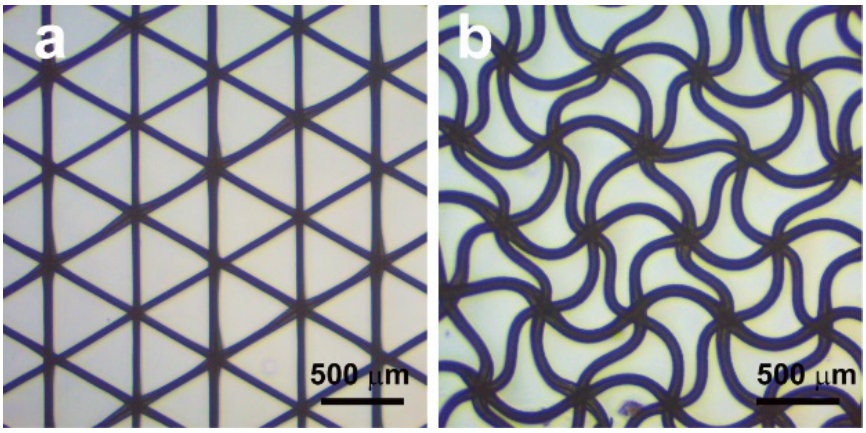

Figure S3. Optical images of a triangular PCL lattice (**a**) before and (**b**) after deformation.

Fabrication of a tetragonal polylactic acid lattice and its reconfigurability

To verify our photochemical reaction-induced expansion theory and extend its application fields, polylactic acid (PLA, Nature works 4032D, USA) was used to fabricate a tetragonal lattice via MEW (Figure S4a). The lattice was subjected to the same treatment as that of the PCL lattice and underwent deformation (Figure S4b). Different from the tetragonal PCL lattice, the deformed PLA lattice (with initial unit cell width of 500 μm) exhibited non-uniform sinusoidal curves. Thus, our proposed strategy can be applied to other UV-initiated graft polymerization material systems.

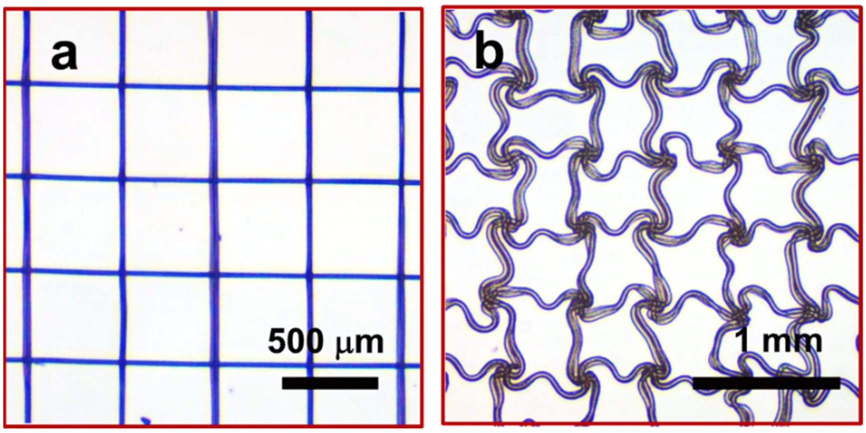

Figure S4. Optical images of a tetragonal PLA lattice (**a**) before and **(b)** after deformation.

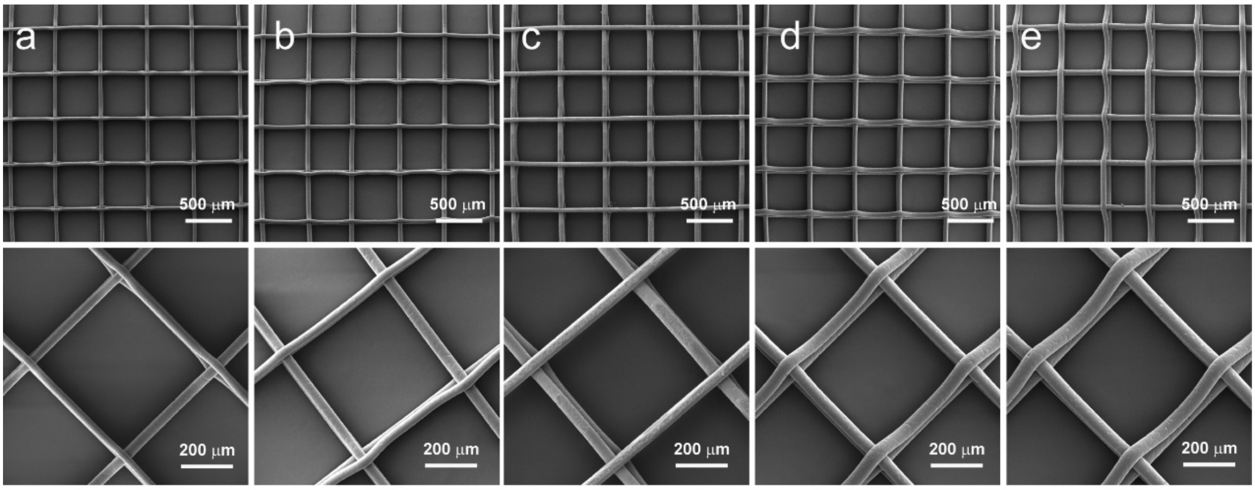

Figure S5. Scanning electron microscopy images of tetragonal PCL lattices fabricated at extrusion pressures of (**a**) 200, (**b**) 300, (**c**) 400, (**d**) 500, and (**e**) 600 kPa.

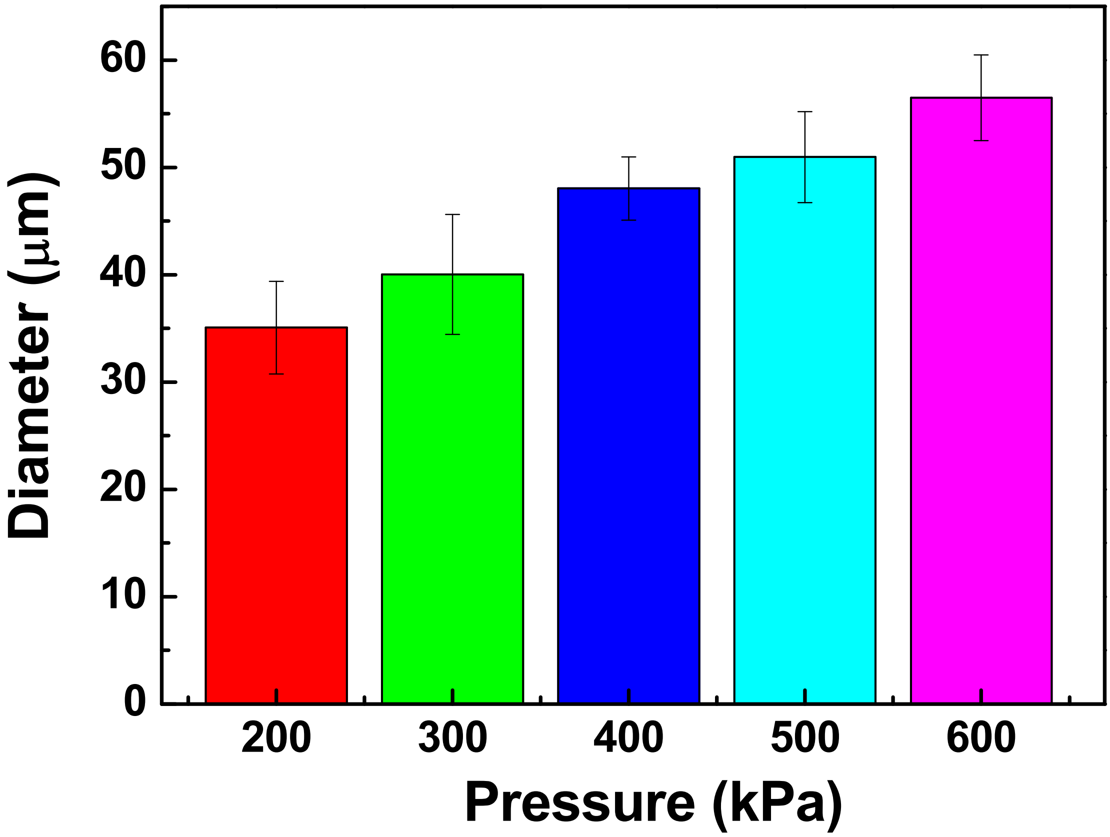

Figure S6. Distribution histogram of PCL beam diameters prepared at extrusion pressures of 200, 300, 400, 500, and 600 kPa.

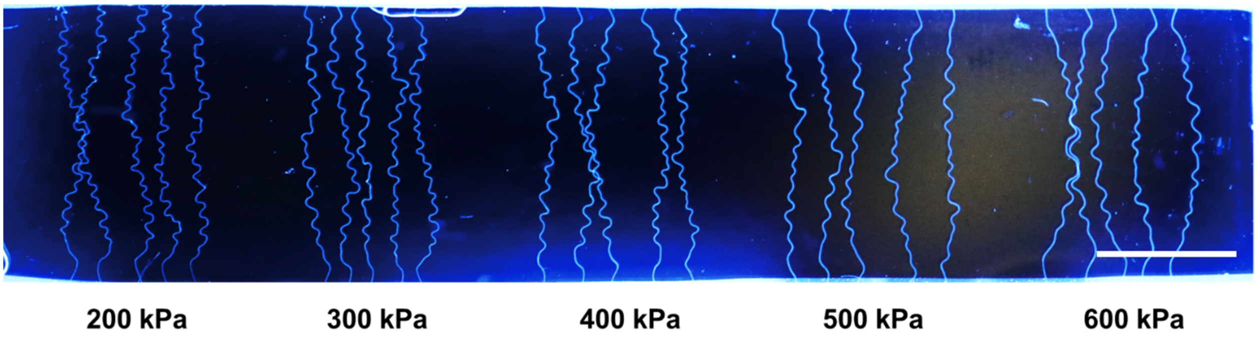

Figure S7. Digital photograph of PCL fibers prepared at extrusion pressures of 200, 300, 400, 500, and 600 kPa after deformation. PCL fibers were initially straight with both ends fastened tightly. Scale bar: 1 cm.

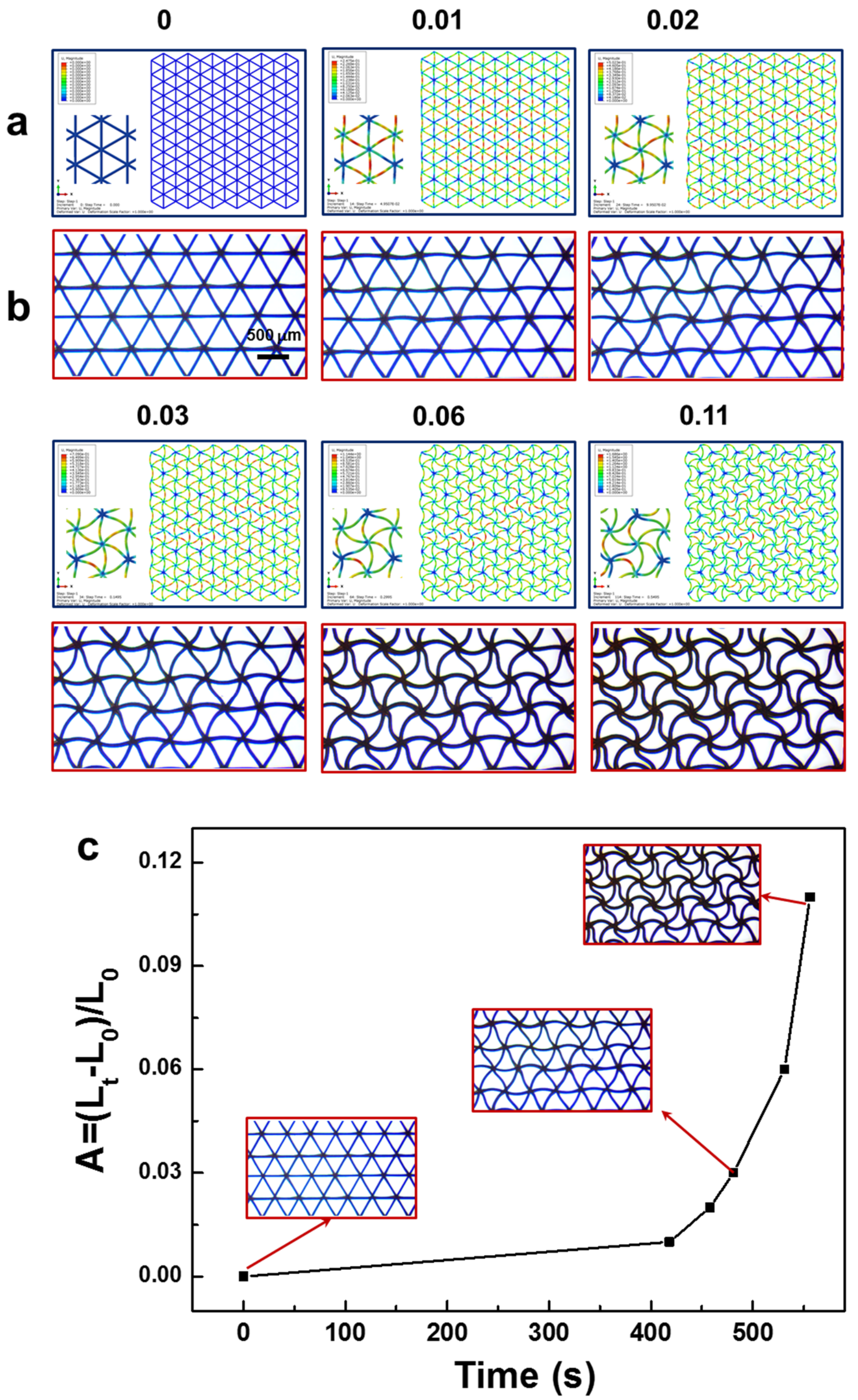

Figure S8. Finite-element modelling of triangular PCL lattices. Geometries of (**a**) simulated and (**b**) experimental PCL lattices with different axial elongation ratios over the deformation period. (**c**) Experimental axial elongation ratio versus time over the deformation period.

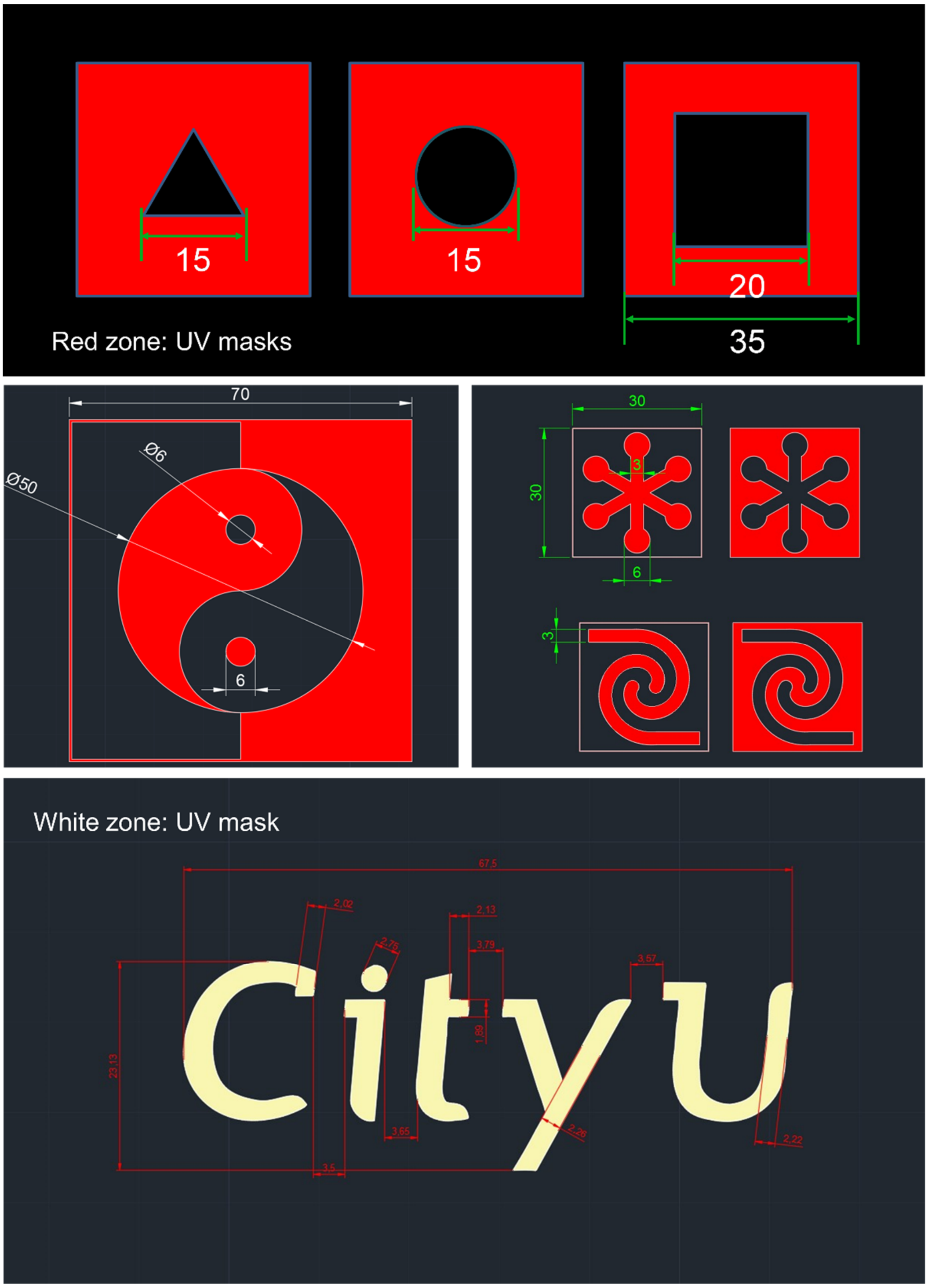

Figure S9. Design drawings of UV masks for controlling domain formation. UV light can't penetrate the red zones (in triangular, round, square, snowflake-shaped, hyperbolic, 'Taiji') and white zone (in 'City U'). (unit: mm)

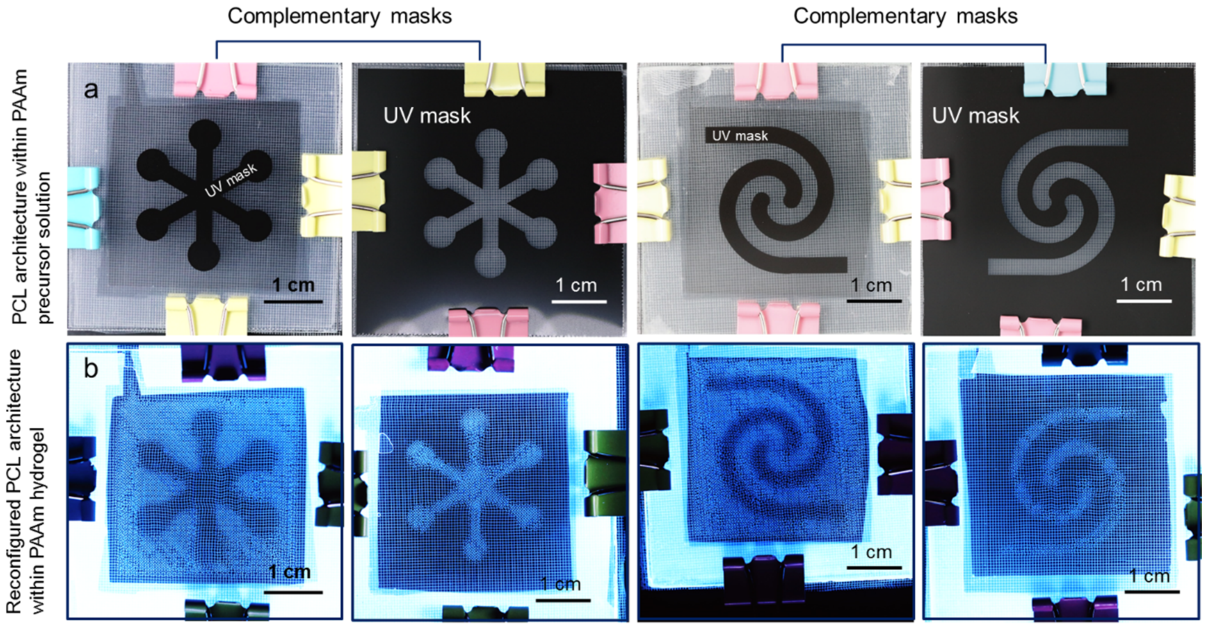

Figure S10. (**a**) Images of the device covered by complementary snowflake-shaped and hyperbolic UV masks. (**b**) Images of the obtained snowflake-shaped and hyperbolic patterns with complementary reconfiguration zones in the PCL architectures.

Illustration of three potential applications

(1) Information steganography

From our current study, the reconfigurable process of PCL lattice can be divided into two distinct stages. In stage I, there are nearly no changes for the PCL lattices. To take advantage of that, PCL lattices are pre-irradiated for a while in Stage I covered by special informative UV masks. As shown in Figure S11, Person A wants to send 'Taiji-information' to Person B. First, Person A exposures the device sealed with PCL tetragonal lattice and PAAm precursor solution by UV light, by controlling the UV exposure time in Stage I (no reconfiguration occurs). Person B receives the preprocessed device and continues to exposure it by UV light. Finally, 'Taiji-information' appears and is received by Person B.

(2) Environmentally adaptive materials

Both the PCL lattice and PAAm precursor solution are sealed in the gap between two transparent glass plates. UV light can penetrate the glass plates and trigger the reconfiguration of the PCL lattice architectures within the PAAm precursor solution (Figure S12(a)). As a result, the reconfigured PCL beams expand volumetrically and bend, leading to the blur in the UV exposure zone (Figure S12(b)). This 'blurred' zone can affect the penetration of visible

light, causing unclear observation to objects behind the UV zone. It may inspire a kind of UV-responsive smart glass, which is controlled by the UV intensity and exposure time.

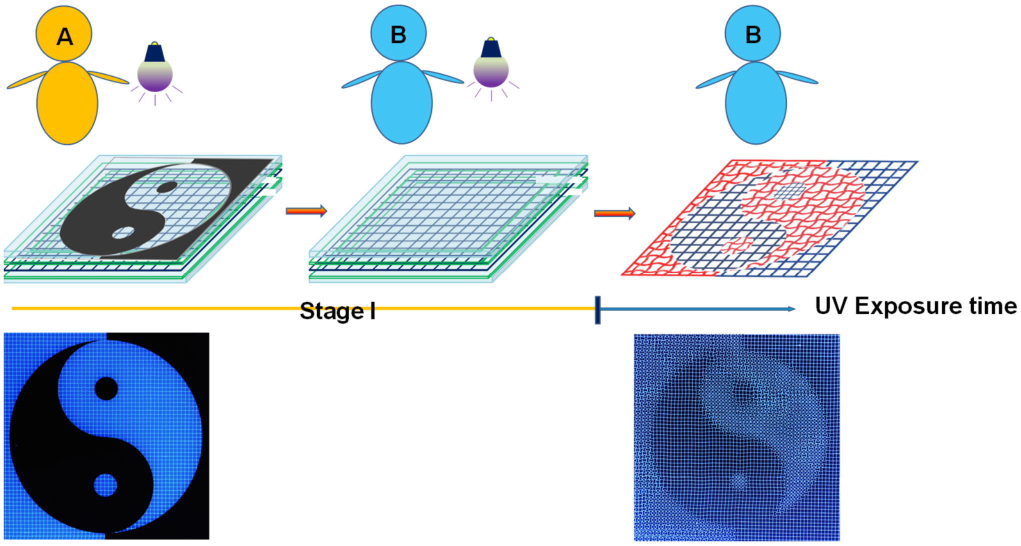

Figure S11. Illustration of a potential application scenario for information steganography with 'Taiji-information' as a model.

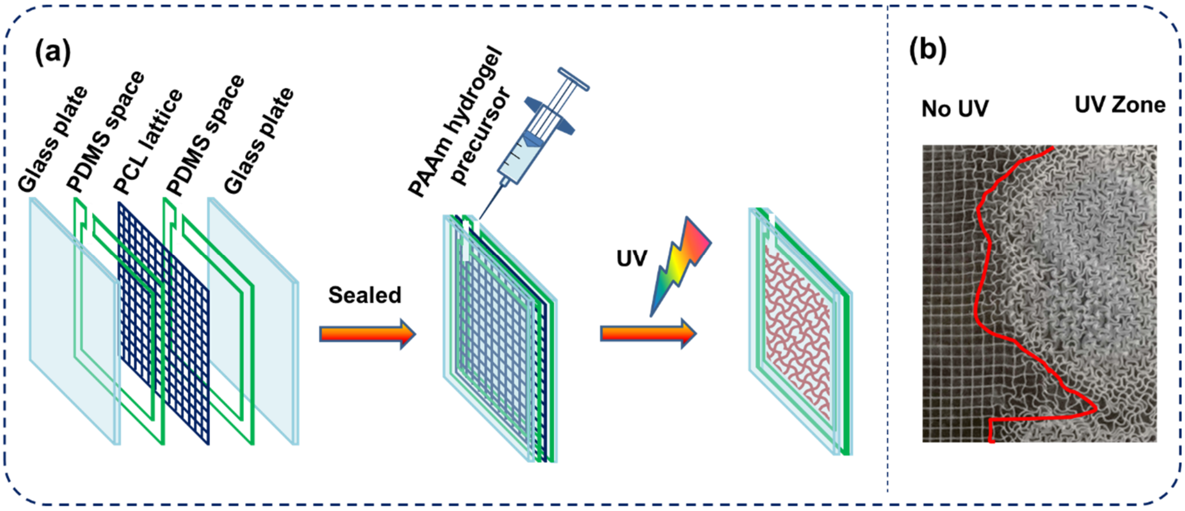

Figure S12. (a) Schematic diagram of the fabrication process for the UV-environmentally adaptive materials, such as a kind of UV-responsive smart glass. (b) An image of a PCL lattice with a blurred zone after UV exposure and a blank zone without UV exposure.

(3) Controlled drug-release systems

PCL beams go through volumetric expansion during the reconfiguration process, accompanying the formation of cracks on their surfaces (Figure S13(a)). If the drug particles are imbedded within the matrix of PCL beams, the expansion process and formation of cracks on the surfaces of PCL beams are beneficial for the generation of release channels for the

drugs. What's more, the generation process of channels can be controlled by the UV exposure time. As a result, we think this phenomenon can be used as a controlled drug-release system (Figure S13(b)).

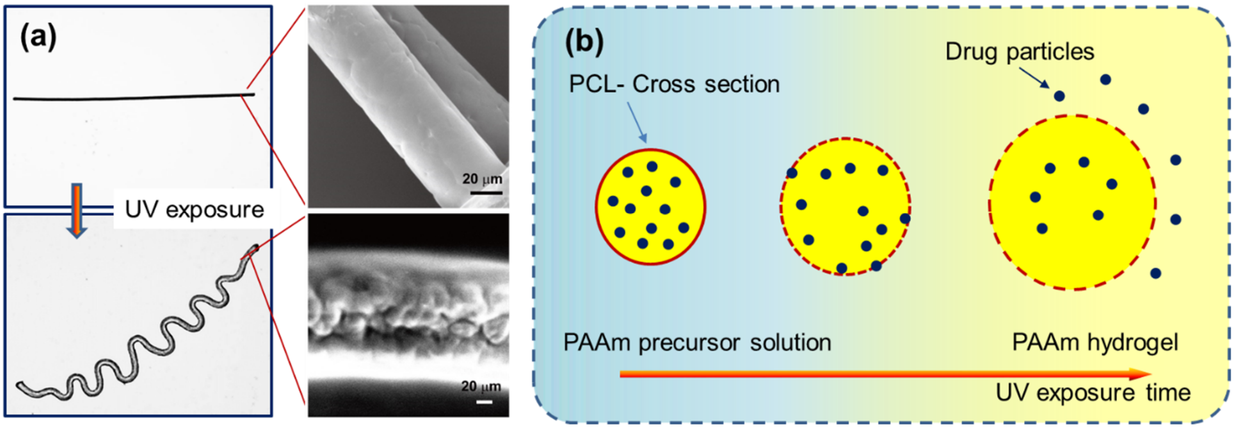

Figure S13. (a) Images of PCL beams and the corresponding surfaces before and after reconfiguration. (b) Schematic diagram of a controlled drug-release systems by using the expansion of PCL beams and cracks formation on the surface of PCL beams.

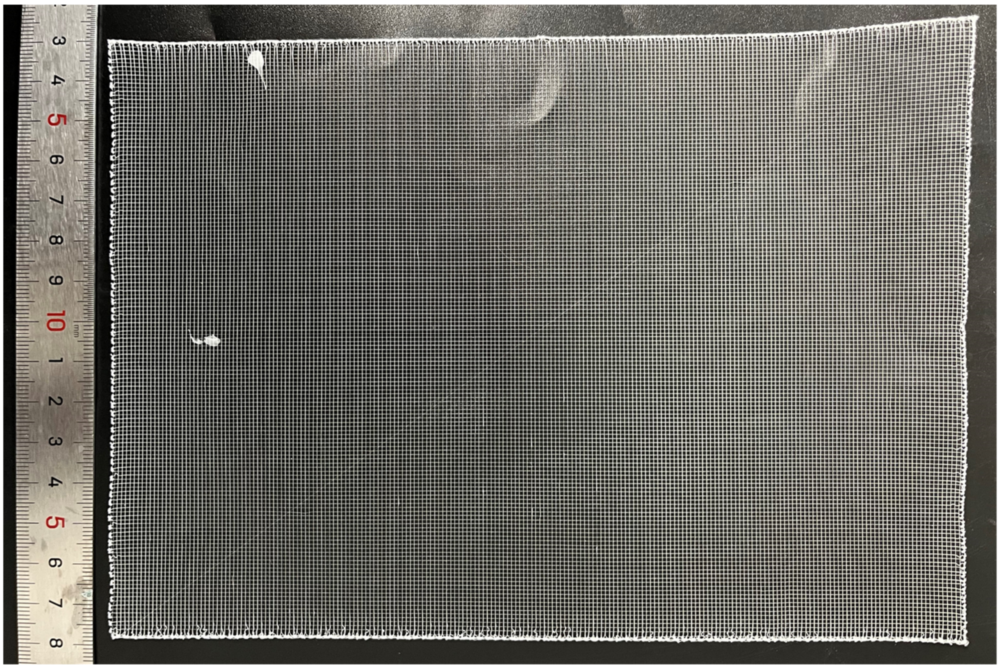

Figure S14. Optical image of a tetragonal PCL lattice with a length of 21 cm.

Table S1 Length–relative error chart of reconfigurable architected materials.

	Beam diameter (D: mm)	Length of lattice (L: mm)	Relative error (D/L) × 100%
Current work	35	210,000	0.017%
2020, ***Science*** [18]			2.5%
2019, ***Nature*** [15]	2.6	1580	0.16%
2021, ***Nature*** [19]	7	10,000	0.07%
2019, ***Adv. Mater.*** [5]	85.7	37,100	0.23%
2015, ***Adv. Mater.*** [25]	200	60,000	0.33%
2017, ***Phys. Rew. Appl.*** [26]	405	150,000	0.27%
2016, ***Adv. Mater.*** [13]	1500	146,890	1.02%
2019, ***PNAS*** [12]	400	165,000	0.24%

Supplementary Movies

Movie S1. Deformation of a tetragonal PCL lattice

Movie S2. Volumetric expansion and curvature generation of PCL beams with lengths of 348.7 μm and 704 μm

Movie S3. Volumetric expansion and curvature generation of a PCL beam with a length of 2091.5 μm

Movie S4. Deformation of a triangular PCL lattice

Movie S5. Deformation of a tetragonal PLA lattice

Movie S6. Deformation control via UV on–off regulation

Movie S7. Finite-element simulation of the Stage II deformation process of a tetragonal PCL lattice

Movie S8. Finite-element simulation of the Stage II deformation process of a triangular PCL lattice
